# ADP-ribose polymer - a novel and general biomarker of human cancers of head & neck, breast, and cervix

**DOI:** 10.1186/1476-4598-9-286

**Published:** 2010-10-30

**Authors:** Rennie O Lakadong, Amal C Kataki, Rajeshwar N Sharan

**Affiliations:** 1 Radiation and Molecular Biology Unit, Department of Biochemistry, North-Eastern Hill University (NEHU), Shillong 793022, India; 2 Dr. B. Barooah Cancer Institute (BBCI), Guwahati, India

## Abstract

**Background:**

Poly-ADP-ribosylation, a reversible post-translational modification of primarily chromosomal proteins, is involved in various cellular and molecular processes including carcinogenesis. ADP-ribose polymer or poly-ADP-ribose adducts are enzymatically added onto or stripped off the target chromosomal proteins during this metabolic process. Due to this, the chromatin superstructure is reversibly altered, which significantly influences the pattern of gene expression. We hypothesize that a decrease in the concentration of total poly-ADP-ribose adducts of peripheral blood lymphocyte (PBL) proteins strongly correlates with the incidence of human cancer.

**Results:**

Using a novel immunoprobe assay, we show a statistically significant (P ≤ 0.001) reduction (~ 42 to 49%) in the level of poly-ADP-ribose adducts of PBL proteins of patients with advanced cancers of head & neck (H & N) region (comprising fourteen distinct cancers at different sites), breast and cervix in comparison to healthy controls.

**Conclusions:**

These findings imply potential utility of the poly-ADP-ribose adducts of PBL proteins as a novel and general biomarker of human cancers with potentials of significant clinical and epidemiological applications.

## Background

Poly-ADP-ribosylation is an enzyme catalyzed reversible post-translational modification of cellular proteins [reviewed in [1-5]]. During this process, ADP-ribose units are transferred onto or stripped off the acceptor or target proteins. Accordingly, the target protein becomes poly-ADP-ribosylated or deribosylated, respectively. The biosynthesis of the homopolymer of ADP-ribose or poly-ADP-ribose adduct(s) is primarily catalysed by the enzyme, poly-ADP-ribose polymerase (PARP), which is ubiquitously present in the nuclei of metabolically active cells [1,5,6]. The enzyme progressively and sequentially catalyses transfer of ADP-ribose units derived from the endogenous nicotinamide adenine dinucleotide (NAD^+^) donors onto acceptor or target proteins creating homopolymeric adducts in a variety of linear and branched architectures. Nuclear proteins, particularly histones, are the primary targets of ribosylation [3-5]. Poly-ADP-ribose glycohydrolase (PARG) is the main enzyme involved in the breakdown of adducts from the protein [1,3,4]. The build up of the adduct on or its breakdown from the chromosomal proteins reversibly alters chromatin superstructure and, thereby, significantly influences the pattern of gene expression [1-5]. Consequently, status of ribosylation of chromosomal proteins is intricately involved in carcinogenesis [4,5]. Previous detailed investigations done in murine model have shown that the metabolic level of the ADP-ribose adduct on total cellular proteins is significantly reduced during different stages of carcinogenesis [7-13]. These systematic studies have revealed the existence of an inverse relationship between carcinogenesis and the total ADP-ribose polymers or poly-ADP-ribose adduct level measured either in cancerous tissue or in peripheral blood lymphocytes (PBL). Therefore, we hypothesize that a decreased quantum of the total adduct of poly-ADP-ribose on human PBL proteins would also correlate with human cancer. In order to test the hypothesis, we have detected and quantified the total concentration of ADP-ribose polymer adducts of PBL proteins in healthy individuals (controls) and cancer patients (cases). PBL was chosen since PBL proteins have been shown to mirror the status of ribosylation of cellular proteins of liver, spleen, ascites cells and other tissues [10]. In addition, a blood based assay of ADP-ribose polymer adducts is the most convenient procedure to perform on humans since it is the most non-invasive medical intervention. An assay using other tissues or biopsies pose practical problems as obtaining the tissue usually requires surgical intervention. Furthermore, the immune system of the body resides in the blood. The PBL are the first responders to foreign invasions across the body. They also communicate with cells as well as cellular matrices of all tissues/organs of the body [14]. This circulating tissue, therefore, qualifies to be the sentinel of the whole body [15]. We have chosen the PBL for this reason as our aim was to identify and establish a general biomarker of cancer, which can be conveniently monitored.

This hypothesis has been tested in total cellular proteins of the PBL of normal human donors (controls) and in patients (cases) with advanced head & neck (H & N), breast or cervical cancers using a novel immunoprobe assay that quantitatively measures the total cellular poly-ADP-ribose polymer [5,7,8].

## Results & Discussion

**Figure 1:**
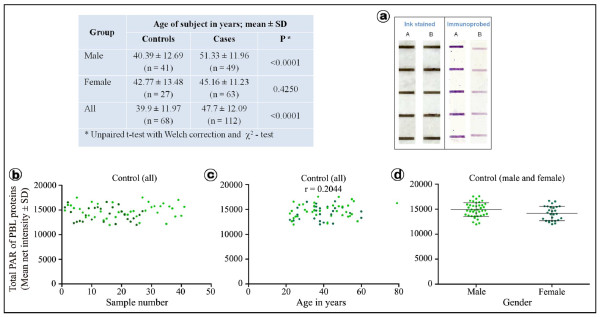
**Quantitative assay of ADP-ribose polymer adducts and analysis of controls**. (**a**) A typical set of results of slot blot immunoprobe assay used in the study. PBL protein (500 ng per slot) samples from a control (A) and a cancer patient (B) were slot blotted on NCM in replicates of five or more. One NCM blot was stained with India ink for total protein (left panel), which also served as the loading control, and its replica slot was immunoprobed for total ADP-ribose polymer (right panel). After concentration correction, quantification of total ADP-ribose polymer of PBL proteins was done as mean net pixel intensity of the immunoprobed slot in arbitrary units (AU). Each sample was independently slotted thrice (3 sets) making at least 15 replicates for each sample (individual). The mean of all replicates for each sample/individual has been plotted as a dot (dark green - female (n = 27); light green - male (n = 41)) in the graphs to highlight the observed individual variance (**b - d**). Linear regression of the controls (r^2 ^= 0.003676, P = 0.6233, n = 68) show that individual value fell within the range of variation (**b**), which was nearly the same when viewed in terms of the age of the individual (**c**). The control values between gender groups (male and female) were statistically (P = 0.0463) identical (**d**). The mean age of male cancer patients (cases) was statistically higher than the control while it was statistically identical in case of females (**inset Table**).

Blood samples were collected from new cancer patients (cases), who had not received any treatment so far, as well as from healthy volunteers (controls) in accordance with the approvals of Institutional Ethics Committees of NEHU and BBCI. The cases comprised male as well as female patients with advanced stages of cancers at 14 different sites of head & neck region (grouped as Ca H & N), breast and cervix (age range 25-80 years). The controls consisted of healthy, male and female volunteers with no known history of cancer (age range 25-80 years). Analysis of age distribution of controls and cases was done using unpaired t-test with Welch correction and χ^2 ^test, which showed that statistically older males (P ≤ 0.0001), but not females (P = 0.4250), made the case group (Figure 1 - inset Table).

Overall, the cancer patients (cases) were statistically older (47.70 years) than the control counterparts (39.90 years) (Figure 1 - inset Table). The PBL protein samples (500 ng per slot) were prepared from blood samples of controls (n = 68) and cases (total n = 112). The cases comprised patients with cancers of 14 different sites grouped as Ca H & N (total n = 66), Ca breast (n = 22) or Ca cervix (n = 24). Samples were subjected to quantitative detection of poly-ADP-ribose adducts using the novel slot blot immunoprobe assay followed by densitometric analysis as described [7,8]. Figure 1 - a shows a typical slot blot stained for total proteins using India ink (left panel) and its immunoprobed replicum (right panel) for five replicate samples from a control subject (A) and a cancer patient (B). The concentration corrected mean net intensity of each control sample has been plotted as a dot to show the distribution of measured poly-ADP-ribose adduct levels in relation to sample number (Figure 1 - b), age of patient (Figure 1 - c) and gender of patient (Figure 1 - d) highlight the range of variation in measured control values in arbitrary units (AU) (mean: 14573 ± 1452 AU; range: 11950 - 17540 AU).

**Figure 2:**
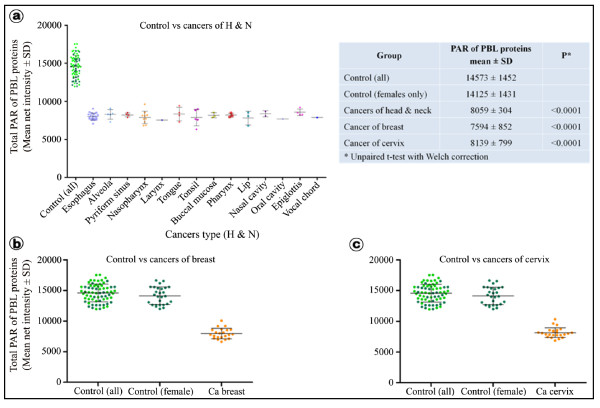
**Quantification of ADP-ribose polymer adducts in cancer patients**. (**a**) The graph shows the levels of ADP-ribose polymer of PBL proteins of each subject as a dot for cancers of different sites in the head and neck region (grouped as cancer of H & N) as compared to all controls (dark green - female (n = 27); light green - male (n = 41)). In all, 14 different types of cancers of H & N were examined in this study; for three such cancers we had only one patient each. Individually all cancers of different sites under H & N category declined significantly below the control value (see details in Table 1). The results from patients with cancers of breast and cervix have been shown in (**b**) and (**c**), respectively. Since these two cancers exclusively afflict females, the plot shows individual values of total ADP-ribose polymer of PBL proteins for all controls (males + females) as well as only female controls. In all three categories of cancers, the reduction in the level of total ADP-ribose polymer of PBL proteins was highly significant (P < 0.0001) when compared with all controls or only female controls (**inset Table**).

Compared to the control group, the total poly-ADP-ribose adduct of PBL proteins of H & N cancer patients showed a statistically significant (P < 0.0001) reduction in cancers of all 14 sites (Figure 2 - a, inset Table).

The overall reduction was approximately 45% with a mean of net intensity of 8060 ± 707.10 AU (range of 6324 - 10318 AU). Cancers of all sites in this group individually (Table 1) as well as collectively (Table 2) showed significant reduction as revealed by Student's t-test and ANOVA, respectively. Similarly, there were significant reductions in the total poly-ADP-ribose adduct of PBL proteins in cancers of breast (Figure 2 - b, inset Table) and cervix (Figure 2 - c, inset Table) in comparison to all controls (males + female controls combined) as well as only female controls. The extent of reduction in the poly-ADP-ribose adduct among the cases was 47% and 43% for breast and cervical cancers, respectively, which are statistically highly significant (P < 0.0001, unpaired t-test with Welch's correction) as shown in the inset table (Figure 2). These results are in line with previous studies done in murine model, which showed negative and statistically significant correlation between total cellular poly-ADP-ribose adduct and carcinogenesis [8-13].

**Table 1:** Poly-ADP-ribose adduct levels in controls and individual cancers of H & N

Category/Type/Site of cancer	Number of cases (n)	Poly-ADP-ribose adduct level (AU) (Mean ± SD)	r^2^	P
**Control**	**68**	**14573 ±1452**		

**All cancer of head and neck (H & N) region**	**66**	**8059 ± 304**		

Cancer of oesophagus	19	8007 ± 463	0.9247	< 0.0001

Cancer of alveolo	6	8173 ± 651	0.9761	< 0.0001

Cancer of pyriform sinus	4	8196 ± 258	0.9759	< 0.0001

Cancer of nasopharynx	10	7890 ± 833	0.9611	< 0.0001

Cancer of larynx	1	7537	NA	NA

Cancer of tongue	4	8340 ± 885	0.893	0.002

Cancer of tonsil	5	7879 ± 1081	0.9713	< 0.0001

Cancer of buccal mucosa	3	8172 ± 327	0.9903	< 0.0001

Cancer of pharynx	3	8177 ± 261	0.9870	< 0.0001

Cancer of lip	3	7801 ±906	0.9869	0.0066

Cancer of nasal cavity	2	8554 ± 440	NA	NA

Cancer of oral cavity	1	7681	NA	NA

Cancer of epiglottis	4	8553 ± 440	0.9849	< 0.0001

Cancer of vocal chord	1	7873	NA	NA

Table showing the analysis of unpaired t test with Welch corrections between total poly-ADP-ribose adduct level of PBL proteins (mean ± SD) in control and each site of cancer of head and neck (H & N) region. The value at each site of cancer was compared to the control separately (NA - not applicable as n was 1(single case)).

**Table 2:** ANOVA of poly-ADP-ribose adduct in controls and cancer of H & N region

Category/Type/Site of cancer	Number of cases (n)	Poly-ADP-ribose adduct level (AU) (Mean ± SD)	r^2^	P
**Control**	**68**	**14573 ± 1452**	0.893	** <**0.0001
		
**Cancers of head & neck region**	**66**	**8059 ± 304**		
		
Cancer of oesophagus	19	8007 ± 463		
		
Cancer of alveolo	6	8173 ± 651		
		
Cancer of pyriform sinus	4	8196 ± 258		
		
Cancer of nasopharynx	10	7890 ± 833		
		
Cancer of larynx	1	7537		
		
Cancer of tongue	4	8340 ± 885		
		
Cancer of tonsil	5	7879 ± 1081		
		
Cancer of buccal mucosa	3	8172 ± 327		
		
Cancer of pharynx	3	8177 ± 261		
		
Cancer of lip	3	7801 ± 906		
		
Cancer of nasal cavity	2	8554 ± 440		
		
Cancer of oral cavity	1	7681		
		
Cancer of epiglottis	4	8553 ± 440		
		
Cancer of vocal chord	1	7873		

Table showing the one-way analysis of variance (ANOVA) between total poly-ADP-ribose adduct of PBL proteins (mean ± SD) of control and cancer of head and neck (H & N).

As this report deals with elucidation of a general biomarker of cancer, the mechanistic aspects of lowering of the level of poly-ADP-ribose adduct of cellular proteins and its likely implications on the biological functions of chromatin, including gene expression, is not being discussed here. These aspects have been reviewed in details recently [5] as well as earlier [4]. It might be prudent to add that in the our considered opinion, the PBL, being the first responders of the body to sense metabolic changes and mount appropriate biological response(s), are the most appropriate cells to look for the alterations in the cellular concentration of the ADP-ribose polymer during carcinogenesis.

The subjects under investigations are likely to be exposed to different and variable etiological factors. Etiological factors could influence both measured poly-ADP-ribose adduct level as well as the course of carcinogenesis. Therefore, we also analysed the influence of three most important etiological factors relevant to the study on poly-ADP-ribose adduct and human cancer. The factors are habits of (a) wet variety of betel nut (BN) chewing - a social etiquette that is strongly associated with cancers, especially to cancer of H & N [reviewed in [16,17]], (b) alcohol consumption [reviewed in [18]] and (c) tobacco usage [reviewed in [19,20]].

**Figure 3:**
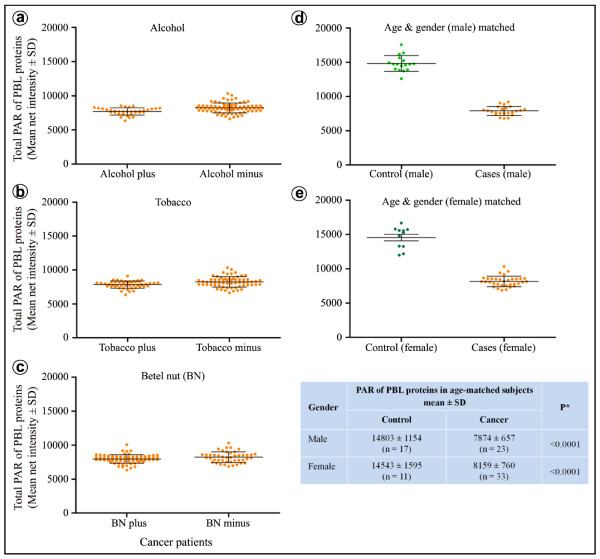
**Effects of selected etiological factors on the cellular level of ADP-ribose polymer adducts in cancer patients**. (**a - c**) The graphs show the effect of three main cancer related etiological factors on the total ADP-ribose polymer of PBL proteins in cancer patients following Spearman rank correlation test and Mann-Whitney rank sum test. The value for each patient is shown as a dot. Cancer patients exposed to alcohol recorded a statistically highly significant (P < 0.0001) reduction in the mean value of ADP-ribose polymer of PBL proteins as compared to patients not exposed to alcohol (**a**). Similarly, patients exposed to tobacco also recorded significantly (P = 0.0033) lower mean ADP-ribose polymer value than those not exposed to tobacco (**b**). In contrast, the wet variety of betel nut chewing habit of cancer patient did not seem to significantly (P = 0.0935) lower the level of ADP-ribose polymer when compared to the non-chewers (**c**). The values of total ADP-ribose polymer of PBL proteins for the age- and gender-matched (male - **d**; female - **e**) controls and cancer patients were also plotted. As is evident, the reductions in the level of ADP-ribose polymer of PBL proteins is highly significant (**inset Table**) suggesting that measure of ADP-ribose polymer of PBL protein is a general biomarker of cancer in humans.

These three factors did not show any statistically significant influence on the level of poly-ADP-ribose adduct in control subjects as revealed by Spearman rank correlation and Mann-Whitney rank sum test (Table 3). However, while BN chewing did not significantly influence the total poly-ADP-ribose adduct of PBL proteins in cancer patients (Figure 3 - c), alcohol consumption (Figure 3 - a) and tobacco usage (Figure 3 - b) were found to lower the level of total poly-ADP-ribose adduct of PBL proteins in cancer patients significantly as compared to the patients without these habits (Table 4).

**Table 3:** Influence of selected etiological factors on poly-ADP-ribose adduct in control subjects

Control subjects	Category	Number of cases (n)	Poly-ADP-ribose adduct level (AU) (Mean ± SD)	P
Gender	Male	41	14870 ± 1405	0.0463*
		
	Female	27	14130 ± 1431	

Tobacco	Yes (+)	9	13879 ± 1617	0.1402
		
	No (-)	59	14605 ± 1418	

Alcohol	Yes (+)	10	14528 ± 1657	0.9379
		
	No (-)	58	14541 ± 1452	

Betel nut	Yes (+)	50	14646 ± 1436	0.9839
		
	No (-)	18	14286 ± 1507	

Diet	Vegetarian	34	14478 ± 1442	0.6194
		
	Non-vegetarian	34	14667 ± 1447	

Table showing the effect of selected cancer-relevant etiological factors on the level of total poly-ADP-ribose adduct of PBL proteins (mean ± SD) in the control subjects. A Mann-Whitney rank sum test was performed to analyze the effect of the etiological factors. None showed any statistically significant influence on the level of total poly-ADP-ribose adduct in PBL except a weakly significant effect of gender; the female control subjects had a slightly lower mean value of poly-ADP-ribose adducts than the male subjects (* - statistically significant).

**Table 4:** Influence of selected etiological factors on poly-ADP-ribose adduct in cancer patients

Cancer patients (Cases)	Category	Number of cases (n)	Poly-ADP-ribose adduct level (AU) (Mean ± SD)	P
Gender	Male	49	8161 ± 643.6	0.0673
		
	Female	63	7982 ± 748.2	

Tobacco	Yes (+)	48	7840 ± 543	0.0033*
		
	No (-)	64	8232 ± 771	

Alcohol	Yes (+)	34	7699 ± 518.1	0.0001*
		
	No (-)	78	8223 ± 718.9	

Betel nut	Yes (+)	68	7963 ± 628.3	0.0935
		
	No (-)	44	8220 ± 791.9	

Diet	Yes (+)	56	8110 ± 707.8	0.6902
		
	No (-)	56	8010 ± 709.2	

Table showing the effect of selected cancer-relevant etiological factors on the level of total poly-ADP-ribose adduct of PBL proteins (mean ± SD) in cancer patients (cases). A Mann-Whitney rank sum test was performed to analyze the effect of the etiological factors. Highly or moderately significant reductions of the total poly-ADP-ribose adduct of PBL proteins were observed in patients exposed to alcohol (compared to alcohol minus patients) and tobacco (compared to tobacco minus patients), respectively. Interestingly, gender, BN and diet did not seem to influence the poly-ADP-ribose adduct level in these patients (* - statistically significant).

In order to further compare the total poly-ADP-ribose adduct of PBL proteins in controls and cases, we matched subjects for age and gender. We obtained 28 controls (11 females and 17 males) and 56 cases (33 females and 23 males) in the age range of 24-65 years (Figure 3 - d, e and inset Table). Again a statistically highly significant (P < 0.0001) reduction (~ 47% in males and ~ 44% in females) in total poly-ADP-ribose adduct of PBL proteins was observed in cancer patients when compared with their age and gender adjusted controls.

## Conclusions

In conclusion, our findings reveal statistically highly significant negative correlation between total poly-ADP-ribose adduct of PBL proteins in advance human cancers of H & N (14 different types), breast and cervix in comparison to healthy controls using a novel immunoprobe assay of poly-ADP-ribose adduct. Based on the preliminary data, we conclude that poly-ADP-ribose adduct of PBL proteins is a useful and general biomarker of cancers in humans. Potentially it can be used as a convenient tool for detection of cancer using the novel immunoprobe assay. The ease with which the assay could be performed along with the results presented in this report support our earlier contention [8,10-13] that the total poly-ADP-ribose adduct of PBL proteins can potentially be used as a tool for mass screening of cancer. We are aware of the fact that further studies on patients in early stages of different cancers and patients undergoing chemo- or radiotherapy, etc. would strengthen the reported findings.

## Methods

### Chemicals

All chemicals were of analytical grade and were used without further purification. All solutions were prepared in sterile ultrapure (Milli-Q) water. Histopaque-1077, phenylmethanesulfonyl fluoride (PMSF) and 0.45μ nitrocellulose membrane (NCM) were purchased from Sigma-Aldrich, USA. RPMI-1640 was purchased from HyClone, Utah, USA and India ink from Rotring Zeichentusche Drawing Ink, Hamburg, Germany. Alkaline phosphatase-conjugated goat anti-rabbit IgG (ALP~IgG) and 5-bromo-4-chloro-3-indolyl phosphate (BCIP)/nitroblue tetrazolium (NBT) solution were purchased from Bangalore Genei, India. Polyclonal antibody (PAb) against natural, heterogeneous ADP-ribose polymers of spleen cells was raised in the laboratory as described earlier [7,8].

### Cancer (case) and control groups of individuals/subjects

The blood samples were collected from new patients visiting Dr. B. Barooah Cancer Institute (BBCI), Guwahati, India in accordance with the Ethics Committee approvals. The informed and consenting (signed) patients, who have not yet been treated for cancer, were recruited for the study following confirmation of cancers (stage III/IV) of different sites in the head & neck (H & N) region, breast and cervix (age range 25-80 years). Detailed information on each individual patient as well as control was recorded, which included age, gender, and other personal data besides family history, history and pattern of betel nut chewing, alcohol consumption and tobacco use, nutritional history, medical history, and radiation exposure history, etc. Different cancers of the H & N included cancer of the oesophagus, tongue, tonsil, nasopharynx, pharynx, buccal mucosa, alveolus, pyriform sinus, larynx, lip, nasal cavity, epiglottis, oral cavity and vocal cord. The control group consisted of normal healthy volunteers with no known history of cancer (age range 25-80 years).

### Isolation of peripheral blood lymphocyte (PBL) and preparation of samples for immunoprobe assay

Blood was collected in a heparinized tube from the subject. PBL was isolated from the collected blood using Histopaque separation medium as described [10]. Briefly, equal volumes of heparinized blood and balanced salt solution (BSS; pH 7.6 containing 5.5 mM anhydrous D-glucose, 5 mM CaCl_2_, 0.098 mM MgCl_2_, 5.4 mM KCl, 0.145 M Tris and 0.14 M NaCl) were mixed and layered over Histopaque. It was centrifuged at 400 × *g *for 40 min at room temperature (RT). The clear lymphocyte layer at the interface was drawn using a Pasteur pipette and washed twice with 3 volumes of BSS at 100 × *g *for 10 min at RT. The final pellet was suspended in 1 ml of RPMI-1640 and stored at -80°C until further use.

To prepare the whole homogenate (WH) of the isolated PBL, the lymphocytes in RPMI-1640 were pelleted down by centrifugation at 250 × *g *for 5 min at RT and washed with ice cold phosphate buffered saline (PBS; 3.2 mM Na_2_HPO_4_, 0.5 mM KH_2_PO_4_, 1.3 mM KCl and 135 mM NaCl, pH 7.4) at 200 × *g *for 10 min at 4°C. One ml of ice cold lysis buffer (20 mM Tris-Cl, pH 8, 10 mM NaCl, 0.5% Triton X-100, 5 mM EDTA and 3 mM MgCl_2_) was added to the pellet, mixed gently, incubated on ice for 30 min, and the lysate was centrifuged at 5000 × *g *for 10 min at 4°C. The protein content of the resulting supernatant, which was used for the assay of poly-ADP-ribose adducts, was estimated by Bradford method using bovine serum albumin (BSA) as a standard. The supernatant was stored at -20°C, whenever required, until further use.

### Immunoprobe assay of poly-ADP-ribose

The assay for total poly-ADP-ribose adduct of the WH proteins of PBL was done by a novel, slot blot immunoprobe assay developed by us, which employs PAb raised against natural, heterogeneous ADP-ribose polymers of mouse spleen cells. The novelty of the assay lies in the fact that it quantitatively measures the total cellular poly-ADP-ribose adducts irrespective of the target protein to which it may be attached. Therefore, the assay reveals the true metabolic level of total cellular poly-ADP-ribose. The assay has been described in details earlier [7,8]. Briefly, slot-blotting was carried out using a Bio-dot microfiltration apparatus (Bio-Rad). Samples were heat inactivated by dipping the sample tubes in a boiling water bath for 5 min. One hundred μl of sample containing 500 ng protein was loaded in each well and slot blotted on NCM under slow and regulated vacuum. The NCM was then immunoprobed for total poly-ADP-ribose adduct of PBL proteins as well as stained with India ink for total cellular proteins as previously described [8]. Immunoprobe assay involved blocking of the NCM by a 5% non-fat dry milk in tris buffered saline (TBS; 20 mM Tris-Cl, pH 7.5, 500 mM NaCl) at RT, incubation in the primary PAb (1:1000) for 1 h at 37°C and incubation in the secondary antibody, ALP~IgG (1:10000), for 1 h at 37°C. Each of these steps was followed by washing twice in TBS and TTBS (TBS containing 0.05% Tween 20) for 5 min each at RT. The color on the NCM was developed by incubating the NCM in 5-bromo-4-chloro-3-indolyl phosphate (BCIP)/nitroblue tetrazolium (NBT) color developer (5-10 min) at RT.

### Quantitative image analysis

Immunoprobed and India ink stained slot blots were scanned (HP Scanjet 7000C) and digitized. The densitometric analysis was done using KDS-ID software (Kodak). Each sample was slot blotted in replicates of five and analysed. The results have been expressed as the mean net pixel intensity of bands (± SD) in arbitrary units (AU) after concentration correction.

### Statistical analysis

An unpaired t-test with Welch's correction was used to analyze the distribution of mean age. A linear regression analysis with 95% confidence bands was performed to examine the poly-ADP-ribose adduct level within the control group. A One way ANOVA was used to compare the poly-ADP-ribose adduct levels between controls and patients with cancers of the H & N region. An unpaired t-test with Welch's correction was used to compare the poly-ADP-ribose adduct levels between controls and patients with cancers of the breast and cervix. Spearman rank correlation test was performed to evaluate the correlation between poly-ADP-ribose adduct and age. χ^2 ^test was used to examine the influence of various factors (age, gender, betel nut, alcohol, tobacco consumptions, etc.) in each of the groups. A Fisher's exact test was applied when necessary. The analysis of influence of different etiological factors in the controls and cases was performed using Mann-Whitney rank sum test. Mann-Whitney rank sum test was also performed to compare the poly-ADP-ribose adduct levels in control and cases matched for age and gender. P value of < 0.05 was taken as statistically significant. In the entire test a two-tailed P value was used.

## Competing interests

Authors declare no competing interest in the reported work. However, RNS holds an Indian patent (IPR/4.18.2/08044) on ADP-ribose polymer/poly-ADP-ribosylation as a cancer biomarker and a potential tool for cancer detection and screening program.

## Authors' contributions

RNS conceived the project, designed the study, analyzed the data and wrote the manuscript in association with ROL. ROL collected samples from control and experimental (cancer patients) subjects, completed questionnaire, carried out the assay and quantification, collated the data, prepared the graphs, etc. ACK coordinated collection of samples from cancer patients at Guwahati. All authors have read and approved the final manuscript.
